# Oral Disease and Caseous Phthisis

**Published:** 1886-12

**Authors:** John S. Smith

**Affiliations:** Lancaster, Pa.


### SELECTIONS.

### Oral Disease and Caseous Phthisis.

BY JOHN S. SMITH, D.D.S., OF LANCASTER, PA.

Mr. S., aged about twenty years, tall of stature, blue eyes, light complexion, light hair. Having graduated at the Mount Joy Soldier's Orphans' School at the age of sixteen years, and for several years subsequently a member of the Reynolds Rifles, of Lancaster, Pa., Co. C., Eighth Regiment National Guard, of Pennsylvania, visited me in November, 1885, for consultation in regard to an ulcer on the right cheek which had bothered him for over four months. His general health was poor, and besides the abscess, which was discharging on the face, he was bothered with a cough, principally at night after lying down; he also complained of pain over the right side of the chest. The man had lost his body weight, and presented an anæmic appearance. He related to me the following history of his case:

About one year before, in 1884, he suffered from chest trouble to some extent, but during the summer following he went to the country, and came back, feeling, as he said, like himself again, having gained considerably in weight. He resumed work again, as an employee in a cork factory in this city. On the occasion of the fall (1885) encampment of the National Guard at Mt. Gretna, he reported with his company for inspection, and while there he had a severe and painful swelling on his right cheek. He reported to the surgeon in charge, and was told to open the gum-boil, which would soon appear on the gum. He suffered excruciating pain for some time, and as a last resort applied a salve made of soap and sugar to the cheek externally, which opened the abscess on the outside. The salve had the tendency to break up the integument of the cheek, and the parts became very much inflamed. Upon his arrival home he placed himself under the care of a physician, who attended him for several months, with but little if any beneficial result.

I made a careful examination of the case, first removing a strip of adhesive plaster from the ulcer, which revealed an unsightly sore, about three-fourths by one-half inch above the border of the jaw and a little back and over the facial artery on the cheek. The ulcer was sunken, with loose skin hanging over the raw surface, which was much inflamed. In the center a large sinus was discharging a thin watery fluid. Upon looking into the oral cavity, it was observed that the teeth of the lower jaw were large and crowded for room. The teeth were all in place, and apparently free from caries, with the exception of several which were slightly decayed, but not to affect their pulps. The wisdom teeth on both sides of the jaw were through the gums, but crowded well back on the ramus of the jaw. While the right side was abscessed externally, the left side around the wisdom tooth was also discharging pus within the cavity of the mouth. This being the general history of the case, the diagnosis was plain.

DIAGNOSIS.

To look at the oral disease from the dento-surgical standpoint, would indicate death of one or more pulps of the teeth. The teeth, by a careful examination under the usual tests, showed living pulps; we must therefore exclude pulpitis, and devitalized pulp. It has been noticed the teeth are crowded for room, and in the eruption of the third molars, the undue pressure exerted in their eruption caused inflammation of the pericdental membrane, and resulting in suppuration and abscess, and necrosis of a portion of the external plate of the alveolar process.

The patient, being strumous, would still add fuel to the flame excited by the crowded condition of the teeth, and pulmonary trouble had sapped the system to some extent of its vitality, which would greatly interfere with nature to throw off the disease without the assistance of the proper remedies. As it was, the ulcerated face and jaw was slow to heal, even after the operation.

TREATMENT.

The removal of the wisdom tooth, which was not an easy matter to accomplish. The wisdom tooth on the left side was discharging pus around the socket, and as the bone was not diseased, nothing more was needed to effect a cure on that side. On the right side other treatment was indicated. Stimulating injections could now be forced through the fistula from the alveoli. Tincture of iodine and diluted carbolic acid were chiefly relied on, used alternately—every day for one week, the iodine being packed (full strength) on cotton wool in alveoli, until the line of demarcation was established. The dead bone was subsequently removed, and all spicula trimmed smooth by the aid of a rose-shaped bur and the dental engine. This completed the treatment within the oral cavity. Attention was now directed to the large ulcerated surface on the cheek, which was still raw and devoid of cuticle. The discharge from the sinus having ceased, the parts soon showed signs of healthy granulation, and with no other dressing than phenol sodique one-half strength during the first week, and subsequently diluted to one-twelfth its original strength, until the scar tissue was completed, which was in about eight weeks after the abscess ceased voiding. The cheek at this time was perfectly healed, having somewhat of a sunken or depressed appearance, but not nearly so much as was expected from the nature and extent of the break of continuity of the soft parts.

As the weather became inclement and changeable during the following January and February, the lung disease became more severe, the patient was unable to leave his room, except at short intervals, when the weather permitted, he ventured out for exercise. The walking would soon tire him, and he was glad to get back to his room. As the disease progressed, the symptoms became more aggravated. Night sweats, and cough, fever, pains in the chest, and the usual characteristic expectoration, emaciation, but no diarrhœa. The appetite did not fail him until within a short time before the middle of February.

When treating the oral disease, I put the patient on a course of iron treatment, and subsequently the syrup of the hypophosphites was administered, which treatment apparently was beneficial. When the night sweats came on the proper remedies were prescribed internally, as well as externally by sponging the skin, which afforded relief.

In February, about the 12th, I suspended my visits, as the oral disease was cured, and he was having no more trouble from that source. I advised the family to call in a physician to take charge of the case, which they did. The patient, however, began to sink, and died in March, about four weeks afterwards, of caseous phthisis.—*Medical and Surgical Reporter.*

